# Larvicidal and Antifeedant Effects of Copper Nano-Pesticides against *Spodoptera frugiperda* (J.E. Smith) and Its Immunological Response

**DOI:** 10.3390/insects13111030

**Published:** 2022-11-07

**Authors:** Afroja Rahman, Sarayut Pittarate, Vivekanandhan Perumal, Julius Rajula, Malee Thungrabeab, Supamit Mekchay, Patcharin Krutmuang

**Affiliations:** 1 Department of Entomology and Plant Pathology, Faculty of Agriculture, Chiang Mai University, Chiang Mai 50200, Thailand; 2 Department of Physiology, Saveetha Dental College and Hospitals, Saveetha Institute of Medical and Technical Sciences, Saveetha University, Chennai 600077, Tamil Nadu, India; 3 Agriculture Technology Research Institute, Rajamangala University of Technology Lanna, Lampang 50200, Thailand; 4 Department of Animal and Aquatic Sciences, Faculty of Agriculture, Chiang Mai University, Chiang Mai 50200, Thailand; 5 Innovative Agriculture Research Center, Faculty of Agriculture, Chiang Mai University, Chiang Mai 50200, Thailand

**Keywords:** *Spodoptera frugiperda*, eco-friendly, nano insecticides, nanotechnology, mortality, toxicity, larvicidal activity, antifeedant activity

## Abstract

**Simple Summary:**

The polyphagous agricultural pest *Spodoptera frugiperda* (J.E. Smith) has a high level of chemical pesticide resistance. This study’s objective is to create and assess the effectiveness of CuO NPs (copper oxide nanoparticles) with a variety of tests against *S. frugiperda* for larvicidal, antifeedant, immunological, and enzymatic activities. Energy dispersive X-ray (EDaX) analysis and a scanning electron microscope (SEM) were used to analyze copper nanoparticles for the identification of physical and chemical properties. The CuO NPs demonstrated high larvicidal and antifeedant activity. The CuO NPs treatment significantly reduced the number of larval hemocytes 24 h after treatment when compared to the control; hemocyte counts and sizes also varied. The levels of larval acetylcholinesterase enzyme levels were decreased with dose-dependent activity after 24 h of treatment with CuO NPs. The current research conclusively shows that CuO NPs have remarkable larvicidal antifeedant activity.

**Abstract:**

This study aimed to synthesize and evaluate the efficacy of CuO NPs (copper oxide nanoparticles) with varying test concentrations (10–500 ppm) against larvicidal, antifeedant, immunological, and enzymatic activities against larvae of *S. frugiperda* at 24 h of treatment. Copper nanoparticles were characterized by using a scanning electron microscope (SEM) and energy dispersive X-ray (EDaX) analysis. The EDaX analysis results clearly show that the synthesized copper nanoparticles contain copper as the main element, and the SEM analysis results show nanoparticle sizes ranging from 29 to 45 nm. The CuO NPs showed remarkable larvicidal activity (97%, 94%, and 81% were observed on the 3rd, 4th, and 5th instar larvae, respectively). The CuO NPs produced high antifeedant activity (98.25%, 98.01%, and 98.42%), which was observed on the 3rd, 4th, and 5th instar larvae, respectively. CuO NPs treatment significantly reduced larval hemocyte levels 24 h after treatment; hemocyte counts and sizes changed in the CuO NPs treatment compared to the control. After 24 h of treatment with CuO NPs, the larval acetylcholinesterase enzyme levels decreased with dose-dependent activity. The present findings conclude that CuO NPs cause remarkable larvicidal antifeedant activity and that CuO NPs are effective, pollution-free green nano-insecticides against *S. frugiperda*.

## 1. Introduction

The agricultural industry has been hit hard in the past few decades due to global warming, land degradation, and ferocious pests and diseases, among other factors, leading to an overwhelming reduction in crop yields [1]. This has raised concerns with the projection of the year 2050 when the need for food to feed the population is estimated to rise significantly. Coupled with that, insect pests have not only become increasingly voracious but resistant to most of the chemicals used against them [2,3,4]. Specifically, *S. frugiperda* (J.E. Smith) (Lepidoptera: Noctuidae) can damage more than 300 plant species, mainly gramineous species, causing significant economic losses to crops such as maize, rice, crabgrass, sorghum, cotton, and vegetable crops, among others [5,6,7,8,9]. This insect has been a native pest of India, Thailand, and China, and subtropics for decades, but it has since spread across the continent, especially in Brazil, the United States, and Argentina [10]. However, in 2016, *S. frugiperda* was found in west Africa and, within a short time, it spread widely across the continent [10,11,12]. In 2018, this invasive pest was discovered in southern India [12] and has since spread throughout Asian countries [13]

Currently, *S. frugiperda* has been spotted in more than 50 major places in Thailand, with the highest population level in six of the western provinces with large corn fields [14,15]. Active research on how to control it has been going on due to its destructive nature and the threat it poses to corn production among other crops [16]. Unfortunately, entomologists are currently facing a major challenge in developing long-term control strategies for insects and pests, especially for *S. frugiperda* [17,18,19,20]. The widespread use of chemical insecticides and pesticides harms non-target species and the green ecosystem, and it has resulted in increasingly growing resistance in the targeted species [4,21,22,23]. A direct effect of synthetic chemicals on human health and non-target species is currently evident [24]. Furthermore, chemical pesticides are lost during application due to volatilization, oxidation, and photolysis, with less than 0.1% of the product being effective against target organisms [25]. To address the above issues, an innovative, updated approach to insect pest control is needed, which will reduce human and environmental risks [26]. Even in the United States, where it originated, controlling this pest has been a daunting task. Aside from the environmental risks that chemical insecticides and pesticides pose, they often build resistance to certain chemicals over time when they are used to manage the *S. frugiperda*. On the other hand, the application of nanotechnology and using it for insect pest control has opened a new path for pest management [27,28,29,30,31,32,33,34]. Nanoparticles have been used as insect control agents for decades, and they provide an alternative and safer insecticidal source for organic farming and IPM programs [35,36].

Nanotechnology is a new strategy to control crop insect pests with several advantages, such as, effective, pollution-free, target-specific, and cheaper [30,37]. According to Amerasan et al. [38], botanical-derived nanoparticles possess strong feeding, fecundity inhibitory, and growth regulatory effects against stored grain insect pests. Furthermore, copper nanoparticles have stronger insecticidal activity than nanoparticles [9,23,33]. According to Shaker et al. [39], using copper nanoparticles showed strong insecticidal activity against cotton leaf worms. In addition, copper nanoparticles can be easily mixed with polymers and are relatively stable in environmental conditions. Since copper is cheaper and more accessible than other nanometals, it is a cost-effective way to control insect pests in agriculture [9,23,33]. However, the effect of copper nanoparticles on lepidopteran insect control has not been completely understood. The present research aims to chemically synthesize and evaluate the larvicidal, antifeedant, immunological, and enzymatic activities against larvae of *S. frugiperda* at 24 h of treatment under laboratory conditions.

## 2. Materials and Methods

### 2.1. Research Area

The current study is carried out at the Insect Pathology Laboratory, Department of Entomology and Plant Pathology, Faculty of Agriculture, Chiang Mai University, Chiang Mai. The relative humidity of Chiang Mai is 71 ± 5%, while the average temperature ranges from 25 to 35 °C [9].

### 2.2. Insects Culture

Fall armyworm *S. frugiperda* larvae were collected from a maize field at Chiang Mai University, Thailand (98.97° E and 18.77° N). This larval culture was kept in plastic containers (19 cm wide, 27 cm long, and 8 cm high) at 26 ± 2 °C, 85% relative humidity, and a photoperiod of 12:12 h (Dark:Light). Baby young corn is fed to the larvae.

### 2.3. Chemical Synthesis of CuO NPs

A chemical reduction process was used to synthesize copper nanoparticles following the method of Khan et al. [40]. A precursor salt was made from copper (II) sulfate pentahydrate, and the capping agent was starch. The solution of copper (II) sulfate pentahydrate is kept in 120 mL of the starch solution and stirred vigorously for 30 min after adding 0.1 M, 120 mL of copper (II) sulfate pentahydrate solution to the mixture. The solution was then stirred continuously with 50 mL 0.2 M ascorbic acid solution. During the 2 h of heating at 80 °C, 30 mL of a 1 M NaOH solution was gradually added to the prepared solution with constant stirring. In the end, the yellowish-brown color of the solution changed from yellow to brown. As part of the experimental procedure, we hung the solution in a dark place overnight, discarded the supernatant, and kept the pellet for use in further experiments.

### 2.4. Characterization of CuO NPs

A scanning electron microscope (SEM, Hitachi, Bangkok, Thailand) and an energy dispersive X-ray spectrometer (EDaX, Hitachi, Bangkok, Thailand) were used to characterize the synthesized nanoparticles’ morphology and chemical composition. For EDaX (Jeol, Tokyo, Japan), the acceleration voltage was 20.0 kV. A tiny copper stub was covered with a double-sided adhesive carbon conductive tape that was sprinkled with dispersed nanoparticles. A gold-sputtering machine (JFC 1500) is then used to coat the sample with gold.

### 2.5. Larvicidal Activity

The Cu NPs were used to evaluate the insect larvicidal activity after 24 h of treatment. Chemically synthesized nanoparticles with different concentrations (10, 100, 300, and 500 ppm) were prepared, and then larvae were dipped individually in the above-mentioned concentration and allowed to air dry for 10–15 min. Then all the larvae were individually transferred to the bioassay container. About 30 larvae per replication (n = 90) were used separately, and each concentration had three replicates. Corn pieces alone were used as a negative control containing 30 larvae, and these were performed in three replicates (n = 90). After 24 h, results are recorded. By using (1), the mortality was calculated, and natural mortality was corrected by using (2), according to Abbott’s [41] formula.

(1) $$\mathrm{Percentage}\;\mathrm{of}\;\mathrm{mortality}=\frac{\mathrm{Number}\;\mathrm{of}\;\mathrm{dead}\;\mathrm{larvae}}{\mathrm{Number}\;\mathrm{of}\;\mathrm{larvae}\;\mathrm{introduced}}\;\times \;100$$

(2) $$\mathrm{Corrected}\;\mathrm{percentage}\;\mathrm{of}\;\mathrm{mortality}=(1-\frac{N\;\mathrm{in}\;T\;\mathrm{after}\;\mathrm{treatment}}{N\;\mathrm{in}\;C\;\mathrm{after}\;\mathrm{treatment}})\;\times \;100$$

The mortality rate was measured after 24 h of treatment and compared to the control. The percentage mortality rate was calculated using Equation (1), and the adjusted percentage mortality was calculated using Abbott’s formula [41], Equation (2), where T is the number of larvae in the treatment groups and C is the number of larvae in the control groups.

### 2.6. Antifeedant Activity

The outer skin of young, fresh maize was peeled off and sliced into 5–6 cm lengths. To get rid of extra dust and microorganisms, corn kernels are first cleaned with tap water and then with double-distilled water. The maize pieces were coated with various concentrations of chemically synthesized nanoparticles (10, 100, 300, and 500 ppm), and they were then left to air dry for 10 to 15 min. Each concentration consisted of three replicates and employed about 30 larvae per replication. In three replicates (n = 90), the nanoparticles free corn pieces were used as a negative control, which contains three replicates and each replicates contains 30 larvae. After 24 h treatments, the antifeedant activity were recorded. Formula 3 was used to compute the feeding deterrence index (FDI).

$$\mathrm{FDI}\;\frac{C-T}{C+T}\;\times \;100$$

where C and T are the weights of control and treated corn eaten by *S. frugiperda*, respectively.

### 2.7. Total Hemocyte Count (THC)

The Tauber–Yeager fluid (NaCl = 4.65 g, KCl = 0.15 g, CaCl_2_ = 0.11 g, gentian violet = 0.005 g, acetic acid = 0.125 m, distilled water = 100 mL) was used to dilute hemolymph to the 0.5 mL threshold. After that, the pipette was gently mixed for 2–3 min. After that the hemocytes were counted using Jones’ formula [42], using an Olympus light microscope (Olympus BXFM, Bangkok, Thailand) set to 40× magnification.

$$\mathrm{Number}\;\mathrm{of}\;\mathrm{hemocytes}/{\mathrm{mm}}^{3}=\frac{X\times \mathrm{dilution}\times 10\times 100}{\mathrm{Number}\;\mathrm{of}\;\mathrm{smallest}\;\mathrm{squares}\;\mathrm{counted}}$$

### 2.8. Acetylcholinesterase Assay

The colorimetric approach previously reported was used to test acetylcholinesterase (AChE) inhibition, Ellman et al. [43]. Monoterpenoids were dissolved in 100% ethanol, and 40 µL of substrate and AChE (100 µL) were combined in a cuvette, followed by DTNB (200 µL) and inhibitor solution (1 mL) in concentrations of 1 mM, 10 mM, 50 mM, and 100 mM. Nonenzymic hydrolysis was compensated for in tests and control assays (without terpenoids). Substrate doses of 1 mM, 2 mM, 5 mM, and 10 mM were employed. Because the results of the second replication were nearly identical to those of the first, each experiment was duplicated only twice. The level of AChE activity was measured at 25 °C using a PharmaSpec UV-1700 Shimadzu Spectrophotometer (American Laboratory Trading (ALT), Bangkok, Thailand) set at 412 nm.

### 2.9. Statistical Analysis

The experimental data were recorded using standard procedures. An analysis of variance and multiple comparison test was performed with the data expressed as (mean ± S.E). According to the Tukey test at *p* < 0.05, statistical values that are separated by the same letter are not substantially different (one-way ANOVA). The statistical analyses were carried out using SPSS version 23.0. (IBM Corp., Armonk, NY, USA, 2015).

## 3. Results

### 3.1. Scanning Electron Microscopy (SEM) Analysis

**Figure 1:**
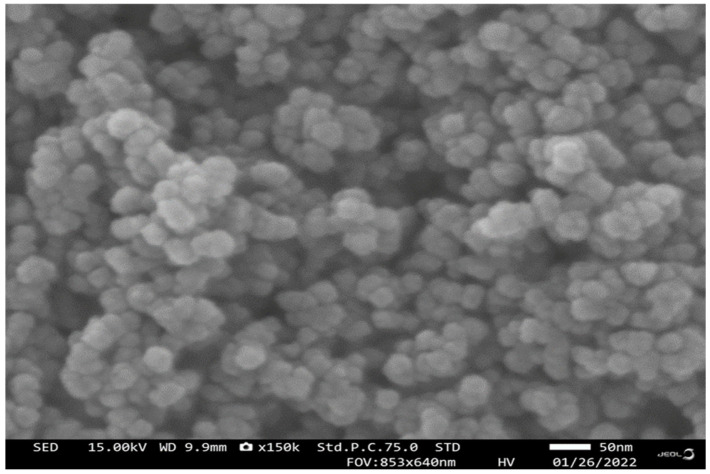
Scanning electron micrographs (SEM) of CuO NPs synthesized by wet chemical reaction, scale 50 nm.

Results of CuO NPs reveal their surface morphology. Copper nanoparticles’ shape is spherical (Figure 1). The nanoparticles’ size range is 29 to 45 nm.

### 3.2. Energy Dispersive X-ray Spectroscopy

**Figure 2:**
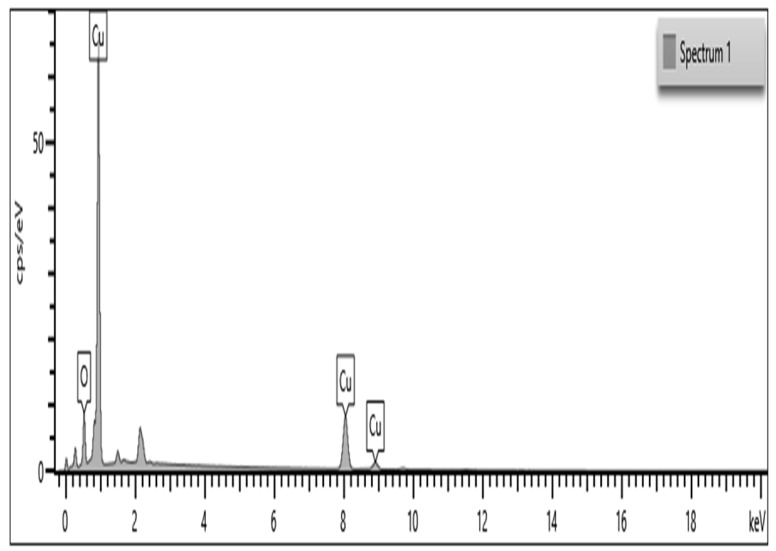
According to the EDaX image, the elementary composition evaluation ranges from 0 to 20 keV. Several elements are shown, and among the elements Cu (copper) is a major particle.

As a result, shown in Figure 2, CuO NPs are a major element according to the EDaX spectrum (Figure 2). A peak at 1, a peak at 8.04, and a peak at 8.94 keV were the strongest. One of the eight necessary plant micronutrients is copper (Cu), which is involved in many enzymatic activities in plants, such as chlorophyll and seed formation. Copper deficiency can make crops more likely to get diseases such as ergot, which can reduce the yield of small grains. Using copper-derived Cu NPs (copper nanoparticles) for insect pest control in the field conditions will result in multiple benefits such as insect control, disease control, increasing enzymatic activity in the plants, and increasing the chlorophyll content.

### 3.3. Larvicidal Effect

**Figure 3:**
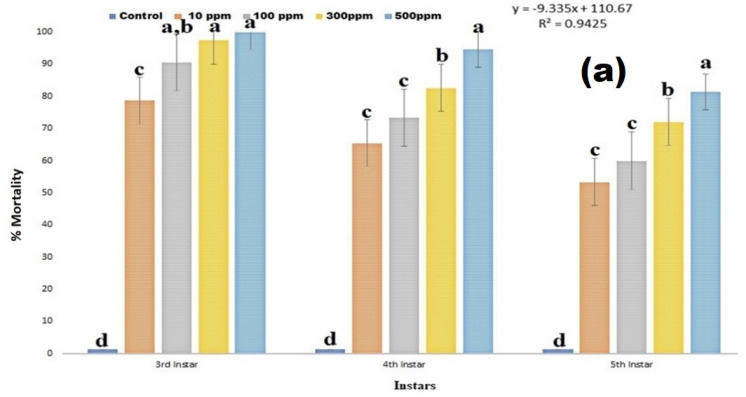

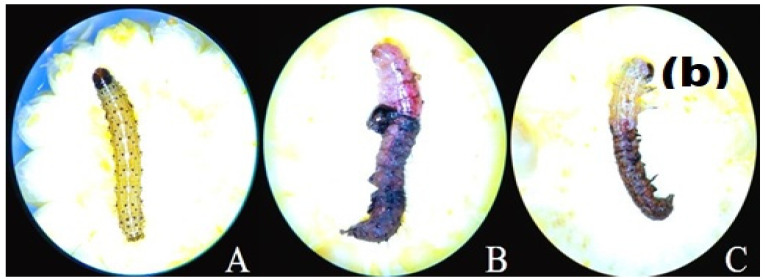
(**a**) is larvicidal activities of copper nanoparticles against 3rd, 4th and 5th instar larvae. (**b**) is morphological changes of control and nanoparticles treated larvae. (**A**). CuO NPs effects on 3rd, 4th, and 5th instar larvae of *S. frugiperda* after 24 h of nanoparticles treatment. (**B**) CuO NPs effects on control and treated *S. frugiperda* larvae (**A**) control larvae (nanoparticles free), (**B**,**C**) nanoparticles treated larvae). According to the Tukey test at *p* ≤ 0.05, statistical values followed by the same letter do not differ significantly (one-way ANOVA).

The larvicidal activity results clearly show that copper nanoparticles caused high larvicidal activity against 3rd instar larvae of *S. frugiperda* and the mortality range is 70–100% (df 4; F_(4,10)_ = 251.632; *p* < 0.01), in 4th instar larvae mortality range is 65–94% (df 4; F_(4,10)_ = 339.000; *p* < 0.01) (Figure 3a,b), in 5th instar larvae mortality range is 53–81% (df 4; F_(4,10)_ = 454.750; *p* < 0.01) (Figure 3a,b). Among the *S. frugiperda* larval instar, the 3rd and 4th instar larvae were highly susceptible to copper nanoparticles treatment.

### 3.4. Antifeedant Effect

**Figure 4:**
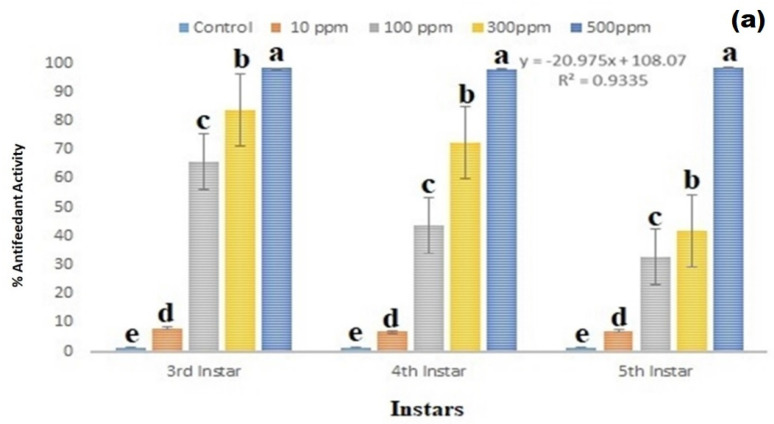

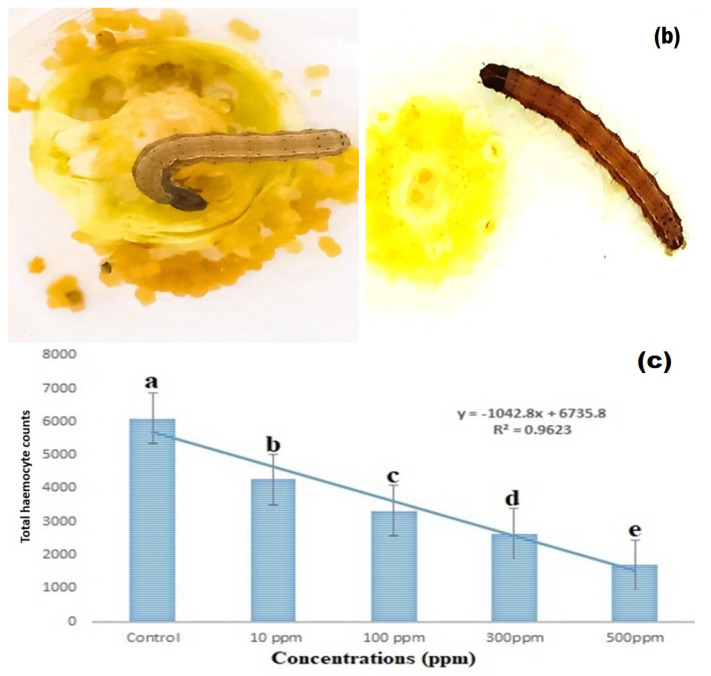
(**a**) CuO NPs antifeedant effect against 3rd, 4th, and 5th instar larvae of *S. frugiperda* after 24 h of nanoparticles treatment. (**b**) CuO NPs antifeedant effect against 3rd, 4th, and 5th instar larvae of *S. frugiperda* after 24 h of nanoparticles treatment. Control (without nanoparticles) and copper nanoparticles treatment. (**c**) Insect larval hemocyte count after copper nanoparticles treatment against 3rd instar larvae of *S. frugiperda*. According to the Tukey test at *p* ≤ 0.05, statistical values followed by the same letter do not differ significantly (one-way ANOVA).

The copper nanoparticles show remarkable antifeedant effects against 3rd, 4th, and 5th instar larvae of *S. frugiperda* after 24 h of nanoparticles treatment in corn. The copper nanoparticles treatment shows remarkable antifeedant effect on 3rd instar larvae of *S. frugiperda*, the antifeedant effect range is 8–98% (df 4; F_(4,10)_ = 4623.617; *p* < 0.01), in 4th instar larval antifeedant effect range is 29–98% (df 4; F_(4,10)_ = 470.181; *p* < 0.01), and in 5th instar larvae antifeedant effect range is 7–98% (df 4; F_(4,10)_ = 2573.571; *p* < 0.01) (Figure 4a,b).

Young corn was treated with copper nanoparticles, and the results showed that the *S. frugiperda* insect larvae did not eat the nanoparticle-treated food (corn). Because of the nanoparticles’ odor and toxicity, *S. frugiperda* insect larvae may avoid corn food. This study clearly shows that the *S. frugiperda* insect pest avoids nanoparticle-treated corn food behaviorally (Figure 4b).

### 3.5. Total Haemocyte Count

**Figure 5:**
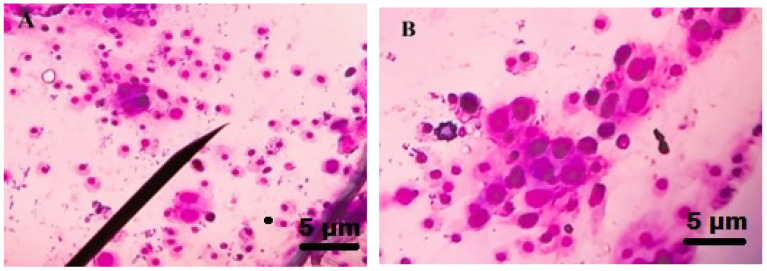
Insect larval hemocyte count after copper nanoparticles treatment against 3rd instar larvae of *S. frugiperda*. (**A**) control (without nanoparticles), (**B**) copper nanoparticles treated.

The results showed that the hemocyte levels were reduced as copper nanoparticle concentration was increased. A higher dose of copper nanoparticles 500 ppm caused a 19.42% reduction in hemocyte count after 24 h of treatment and was statistically different compared to the control (df 4; F_(4,10)_ = 265.073; *p* ≤ 0.01) (Figure 4c and Figure 5). The nanoparticles-treated *S. frugiperda* larval hemocyte sizes were bigger compared to control group.

### 3.6. Acetylcholinesterase Enzyme

**Figure 6:**
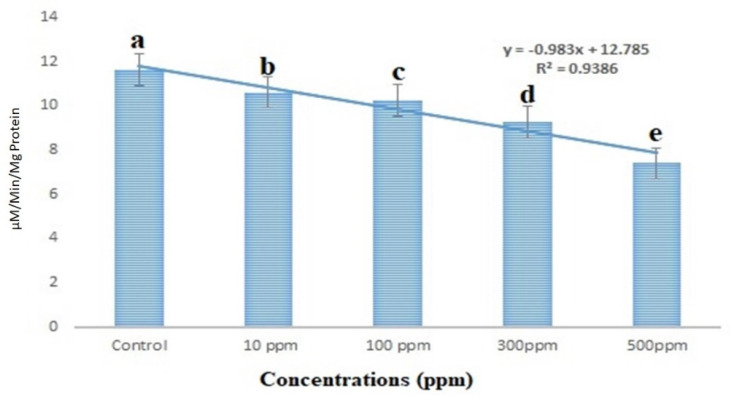
Acetylcholinesterase activity after the treatment with copper nanoparticles against 3rd instar larvae of *S. frugiperda*. According to the Tukey test at *p* ≤ 0.05, statistical values followed by the same letter do not differ significantly (one-way ANOVA).

The *S. frugiperda* larval acetylcholinesterase activity was shown to be lowered by 20.37% at a lower concentration of 10 ppm, according to the results. On the other hand, higher concentrations (500 ppm) revealed a 60.25% reduction in acetylcholinesterase enzyme activity (Figure 6). Acetylcholinesterase activity was statistically different from the control at all treated concentrations (df 4; F_(4,10)_ = 106.408; *p* < 0.01).

## 4. Discussion

In the present studies, we synthesized the CuO nanoparticles using starch as a binding agent and copper sulfate as a primary source. The synthesized nanoparticles were characterized using SEM and EdaX analysis. The copper nanoparticles SEM analysis shows that the nano-particle size range is 29–45 nm and it is spherical in nature. The EDaX analysis results show that copper nanoparticles contain copper as a major element in the chemical-synthesized CuO nanoparticles. Similarly, Vivekanandhan et al. [33] reported that entomopathogenic fungi-derived silver nanoparticles show a similar size to the nanoparticles, and their particles were highly effective against disease-transmitting mosquito vectors *Aedes aegypti*, *Anopheles stephensi* and *Culex quinquefasciatus*.

Currently, several metal nanoparticles were synthesized using different methods such as physically, chemically, and biologically derived metal nanoparticles, but the mode of action of cooper nanoparticle in insect pests is not completely understood [44,45,46]. The nanoparticles can pass through epithelial and endothelial cells using the transcytosis process [47]. Due to this inherent property, nanoparticles are easily able to penetrate dendrites, axons, blood vessels, and lymphatic vessels, which results in oxidative stress [48]. The present studies show that chemically synthesized copper nanoparticles caused high larvicidal activity after 24 h of the treatment. Among the larval stages, the 3rd instar larvae are highly susceptible to copper nanoparticles. Compared to 5th instar larvae, the 4th instar larvae were highly susceptible to nanoparticles. Similar to our studies, Pittarate et al. [9] reported that the chemical synthesized zinc oxide nanoparticles showed remarkable insecticidal efficacy against larvae, pupae, and adults of the *S. frugiperda* insect pest. Several bodily abnormalities were also noticed during the insect’s life cycle in the *S. frugiperda* insect pest. Additionally, the females’ fertility was significantly impacted.

Previous findings demonstrated that *M*. *robertsii*-mediated CuNPs are extremely toxic to the targeted insect pests (*An*. *stephensi*, *Ae*. *aegypti*, *Cx*. *quinquefasciatus*, *T*. *molitor*), but less hazardous to non-target organisms (*A*. *salina, A*. *nauplii, E*. *eugeniae*, and *E*. *andrei*) [49]. The rice weevil, *Sitophilus oryzae* (L.), was reported to have 100 % mortality in adults’ nanoparticles [50]. According to Ki et al. [51], nano-silver-treatment caused high insecticidal activity against *Tinea pellionella* (L.) larvae. Nanostructured alumina (NSA dust) is highly toxic to *R*. *dominica* and *S*. *oryzae* adult insect pest, where 95% mortality was observed after 3 days of nanoparticles treatment. *S*. *oryzae* adults are highly susceptible to NSA [52]. Similarly, in Murugan et al. [53], AgNPs synthesized from *C*. *scalpelliformis* and *C*. *agardh* caused 80% mortality against 1st–4th instar larvae of *Cx*. *quinquefasciatus* with minimal concentrations.

The present study shows that chemically synthesized copper nanoparticles caused high antifeedant activity against the 3rd, 4th, and 5th instar larvae of *S. frugiperda*. Similar to our studies, larvae of *Spodoptera litura* treated with nanoparticles caused remarkable insecticidal activity and developmental changes in insect larvae [54]. Similarly, in this experiment, the total larval period was prolonged when compared to the control. Another study from the same laboratory showed that iron nanoparticles, zinc nanoparticles, cadmium nanoparticles, copper nanoparticles, and lead nanoparticles delayed larval growth in lepidopteran larvae [55,56,57,58]. In addition, larvae in high-metal diets may need more energy for metal-detoxification, which is caused by metals entering into biochemical reactions they are not normally involved in [59,60]. Insect midguts serve as the main sites of absorption and play a significant role in metabolic activity [61]. In the digestive tract, metal interferences accumulate in gut cells, preventing them from entering the bloodstream [62].

Previous research has reported malformations in larvae, pupae, and adults when exposed to nanoparticles. Moreover, Zn NPs and silica nanoparticles have been observed to cause deformations in insects [9,63]. The present studies clearly show that copper nanoparticle treatments reduced the total haemocyte counts and acetylcholinesterase enzyme levels in the larvae of *S. frugiperda* after 24 h. The dose-dependent activity was observed in the hemocyte levels. Similarly, Vivekanandhan et al. [64] reported that insect pathogenic fungi conidia treatment reduced the *S*. *litura* larval hemocyte counts and acetylcholinesterase enzyme levels with dose-dependent activity. CuO NPs are an important class of nanomaterials for a variety of applications (medical, environmental, and industrial), and a few researchers have observed that copper nanoparticles pose potential dangers to non-target animals and the green environment. More research is needed on the physiochemical properties of CuO NPs, concentration, mechanism of action, and non-target toxicity of CuO NPs [65,66,67,68].

## 5. Conclusions

Chemically synthesized copper nanoparticles caused remarkable larvicidal and antifeedant effects as observed against *Spodoptera frugiperda* larvae after 24 h post-treatment. The larval mortality and antifeedant effects increased as the test concentrations increased. The copper nanoparticles-treated larval hemocyte count and acetylcholinesterase enzyme levels were significantly decreased after 24 h of treatment. *S. frugiperda* was also killed by chemically synthesized copper nanoparticles at minimal concentrations with excellent larvicidal and antifeedant activity.

## Data Availability

During the present research entities, the datasets gathered and generated from the analysis after extraction/separation/isolation of compound emodin and evaluated biological results are available from the corresponding author upon reasonable request.

## References

[1] Rosegrant M.W., Ringler C., Sulser T.B., Ewing M., Palazzo A., Zhu T., Nelson G.C., Koo J., Robertson R., Msangi S. (2009). Agriculture and Food Security under Global Change: Prospects for 2025/2050.

[2] Dubovskiy I.M., Whitten M.M.A., Yaroslavtseva O.N., Greig C., Kryukov V.Y., Grizanova E.V., Mukherjee K., Vilcinskas A., Glupov V.V., Butt T.M. (2013). Can Insects Develop Resistance to Insect Pathogenic Fungi?. PLoS ONE.

[3] Zhang D.D., Xiao Y.T., Xu P.J., Yang X.M., Wu Q.L., Wu K.M. (2021). Insecticide resistance monitoring for the invasive populations of fall armyworm, *Spodoptera frugiperda* in China. J. Integr. Agric..

[4] Vivekanandhan P., Thendralmanikandan A., Kweka E.J., Mahande A.M. (2021). Resistance to temephos in *Anopheles stephensi* larvae is associated with increased cytochrome P450 and α-esterase genes overexpression. Int. J. Trop. Insect Sci..

[5] Johnson S.J. (1987). Migration and the life history strategy of the fall armyworm, *Spodoptera frugiperda* in the Western Hemisphere. Int. J. Trop. Insect. Sci..

[6] Pogue G.M. (2005). A world revision of the genus *Spodoptera* Guenee (Lepidoptera: Noctuidae). Mem. Am. Entomol. Soc..

[7] Nagoshi R.N. (2009). Can the amount of corn acreage predict fall armyworm (Lepidoptera: Noctuidae) infestation levels in nearby cotton?. J. Econ. Entomol..

[8] Day R., Abrahams P., Bateman M., Beale T., Clottey V., Cock M., Colmenarez Y., Corniani N., Early R., Godwin J. (2017). Fall armyworm: Impacts and implications for Africa. Outlooks Pest Manag..

[9] Khooshe-Bast Z., Sahebzadeh N., Ghaffari-Moghaddam M., Mirshekar A. (2016). Insecticidal effects of zinc oxide nanoparticles and *Beauveria bassiana* TS11 on *Trialeurodes vaporariorum* (Westwood, 1856) (Hemiptera: Aleyrodidae). Acta Agric. Slov..

[10] Clark P.L., Molina-Ochoa J., Martinelli S., Skoda S.R., Isenhr D.J., Lee D.J., Krumm J.T., Foster J.E. (2007). Population variation of the fall armyworm, *Spodoptera frugiperda*, in the Western Hemisphere. J. Insect Sci..

[11] Bateman M.L., Day R.K., Luke B., Edgington S., Kuhlmann U., Cock M.J.W. (2018). Assessment of potential biopesticide options for managing fall armyworm (*Spodoptera frugiperda*) in Africa. J. Appl. Entomol..

[12] Sisay B., Simiyu J., Malusi P., Likhayo P., Mendesil E., Elibariki N., Wakgari M., Ayalew G., Tefera T. (2018). First report of the fall armyworm, *Spodoptera frugiperda* (Lepidoptera: Noctuidae), natural enemies from Africa. J. Appl. Entomol..

[13] CABI (2019). Spodoptera frugiperda (fall armyworm). CABI: Invasive Species Compendium.

[14] Nelly N., Lina E.C., Hamid H., Yunisman Y. (2021). Distribution and genetic diversity of *Spodoptera frugiperda* JE Smith (Noctuidae: Lepidoptera) on maize in West Sumatra, Indonesia. Biodiversitas J. Biol. Divers..

[15] Bahadur L.C., Bikram A. (2019). Fall armyworm (*Spodoptera frugiperda*): A threat to food security for south Asian country: Control and management options: A review. Farming Manag..

[16] Bhusal S., Chapagain E. (2020). Threats of fall armyworm (*Spodoptera frugiperda*) incidence in Nepal and it’s integrated management-A review. Agric. Nat. Resour..

[17] Hassan A. (2012). Effects of mineral nutrients on physiological and biochemical processes related to secondary metabolites production in medicinal herbs. Med. Aromat. Plant Sci. Biotechnol..

[18] Norris E.J., Johnson J.B., Gross A.D., Bartholomay L.C., Coats J.R. (2018). Plant essential oils enhance diverse pyrethroids against multiple strains of mosquitoes and inhibit detoxification enzyme processes. Insects.

[19] Azeem M., Zaman T., Tahir M., Haris A., Iqbal Z., Binyameen M., Nazir A., Shad S.A., Majeed S., Mozūraitis R. (2019). Chemical composition and repellent activity of native plants essential oils against dengue mosquito, *Aedes aegypti*. Ind. Crops Prod..

[20] O’Neal S.T., Johnson E.J., Rault L.C., Anderson T.D. (2019). Vapor delivery of plant essential oils alters pyrethroid efficacy and detoxification enzyme activity in mosquitoes. Pestic. Biochem. Physiol..

[21] Desneux N., Decourtye A., Delpuech J.M. (2007). The sublethal effects of pesticides on beneficial arthropods. Annu. Rev. Entomol..

[22] Vivekanandhan P., Usha-Raja-Nanthini A., Valli G., Subramanian S.M. (2020). Comparative efficacy of *Eucalyptus globulus* (Labill) hydrodistilled essential oil and temephos as mosquito larvicide. Nat. Prod. Res..

[23] Crow J.F. (1957). Genetics of insect resistance to chemicals. Annu. Rev. Entomol..

[24] Rattan R.S. (2010). Mechanism of action of insecticidal secondary metabolites of plant origin. Crop. Prot..

[25] Athanassiou C.G., Kavallieratos N.G., Benelli G., Losic D., Rani P.U., Desneux N. (2018). Nanoparticles for pest control: Current status and future perspectives. J. Pest. Sci..

[26] Liang Y., Guo M., Fan C., Dong H., Ding G., Zhang W., Tang G., Yang J., Kong D., Cao Y. (2017). Development of novel ure-ase-responsive pendimethalin microcapsules using silica-IPTS-PEI as controlled release carrier materials. ACS Sustain. Chem. Eng..

[27] Baruah S., Dutta J. (2009). Nanotechnology applications in pollution sensing and degradation in agriculture: A review. Environ. Chem. Lett..

[28] Kumar R., Sharon M., Choudhary A.K. (2010). Nanotechnology in agricultural diseases and food safety. J. Phytol..

[29] Sooresh A., Kwon H., Taylor R., Pietrantonio P., Pine M., Sayes C.M. (2011). Surface functionalization of silver nanoparticles: Novel applications for insect vector control. ACS. Appl. Mater. Interfaces.

[30] Barik T.K., Kamaraju R., Gowswami A. (2012). Silica nanoparticle: A potential new insecticide for mosquito vector control. Parasitol Res..

[31] Xie Y., Wang B., Li F., Ma L., Ni M., Shen W., Hong F., Li B. (2014). Molecular mechanisms of reduced nerve toxicity by titanium dioxide nanoparticles in the phoxim-exposed brain of *Bombyx mori*. PLoS ONE.

[32] Roni M., Murugan K., Panneerselvam C., Subramaniam J., Nicoletti M., Madhiyazhagan P., Dinesh D., Suresh U., Khater H.F., Wei H. (2015). Characterization and biotoxicity of *Hypnea musciformis*-synthesized silver nanoparticles as potential eco-friendly control tool against *Aedes aegypti* and *Plutella xylostella*. Ecotoxicol. Environ. Saf..

[33] Vivekanandhan P., Deepa S., Kweka E.J., Shivakumar M.S. (2018). Toxicity of *Fusarium oxysporum*-VKFO-01 derived silver nano-particles as potential inseciticide against three mosquito vector species (Diptera: Culicidae). J. Clust. Sci..

[34] Parthiban E., Ramachandran M., Jayakumar M., Ramanibai R. (2019). Biocompatible green synthesized silver nanoparticles impact on insecticides resistant developing enzymes of dengue transmitted mosquito vector. SN Appl. Sci..

[35] Benelli G., Lukehart C.M. (2017). Applications of green-synthesized nanoparticles in pharmacology, parasitology and entomology. J. Clust. Sci..

[36] Banumathi B., Vaseeharan B., Rajasekar P., Prabhu N.M., Ramasamy P., Murugan K., Canale A., Benelli G. (2017). Exploitation of chemical, herbal and nanoformulated acaricides to control the cattle tick, *Rhipicephalus* (*Boophilus*) microplus—A review. Vet. Parasitol..

[37] Benelli G. (2018). Gold nanoparticles–against parasites and insect vectors. Acta Trop..

[38] Amerasan D., Nataraj T., Murugan K., Panneerselvam C., Madhiyazhagan P., Nicoletti M., Benelli G. (2016). Myco-synthesis of silver nanoparticles using *Metarhizium anisopliae* against the rural malaria vector *Anopheles culicifacies* Giles (Diptera: Culicidae). J. Pest Sci..

[39] Shaker A.M., Zaki A.H., Abdel-Rahim E.H.F., Khedr M.H. (2017). Photocatalytic Degradation of Carbamate Pesticide (methomyl) Using Synthesized TiO2 Nanoparticles against the Cotton Leafworm *S. littoralis*. Egypt. Acad. J. Biol. Sci..

[40] Khan A., Rashid A., Younas R., Chong R. (2016). A chemical reduction approach to the synthesis of copper nanoparticles. Int. Nano Lett..

[41] Abbott W.S. (1925). A method of computing the effectiveness of an insecticide. J. Econ. Entomol..

[42] Jones J.C. (1962). Current concepts concerning insect hemocytes. Am. Zool..

[43] Ellman G.L., Courtney K.D., Andres V., Featherstone R.M. (1961). A new and rapid colorimetric determination of acetylcholinesterase activity. Biochem. Pharmacol..

[44] Poopathi S., De Britto L.J., Praba V.L., Mani C., Praveen M. (2015). Synthesis of silver nanoparticles from Azadirachta indica—A most effective method for mosquito control. Environ. Sci. Pollut. Res..

[45] Oberdörster G., Oberdörster E., Oberdörster J. (2005). Nanotoxicology: Discipline evolving from studies of ultrafine particles. Environ. Health Perspect..

[46] Yamanaka Y.J., Leong K.W. (2008). Engineering strategies to enhance nanoparticle-mediated oral delivery. J. Biomater. Sci. Polym. Ed..

[47] Maynard A.D. (2007). Nanotoxicology: Laying a firm foundation for sustainable nanotechnologies. Nanotoxicology.

[48] Debnath N., Das S., Seth D., Chandra R., Bhattacharya S.C., Goswami A. (2011). Entomotoxic effect of silica nanoparticles against *Sitophilus oryzae* (L.). J. Pest. Sci..

[49] Vivekanandhan P., Swathy K., Thomas A., Kweka E.J., Rahman A., Pittarate S., Krutmuang P. (2021). Insecticidal efficacy of microbial-mediated synthesized copper nano-pesticide against insect pests and non-target organisms. Int. J. Environ. Res. Public Health.

[50] Stadler T., Buteler M., Weaver D.K., Sofie S. (2012). Comparative toxicity of nanostructured alumina and a commercial inert dust for *Sitophilus oryzae* (L.) and *Rhyzopertha dominica* (F.) at varying ambient humidity levels. J. Stored Prod. Res..

[51] Ki H.Y., Kim J.H., Kwon S.C., Jeong S.H. (2007). A study on multifunctional wool textiles treated with nano-sized silver. J. Mater. Sci..

[52] Yasur J., Rani P.U. (2015). Lepidopteran insect susceptibility to silver nanoparticles and measurement of changes in their growth, development and physiology. Chemosphere.

[53] Murugan K., Benelli G., Panneerselvam C., Subramaniam J., Jeyalalitha T., Dinesh D., Nicoletti M., Hwang J.S., Suresh U., Madhiyazhagan P. (2015). *Cymbopogon citratus*-synthesized gold nanoparticles boost the predation efficiency of copepod Mesocyclops aspericornis against malaria and dengue mosquitoes. Exp. Parasit..

[54] Vivekanandhan P., Bedini S., Shivakumar M.S. (2020). Isolation and identification of entomopathogenic fungus from Eastern Ghats of South Indian forest soil and their efficacy as biopesticide for mosquito control. Parasitol. Int..

[55] Rouhani M., Samih M.A., Kalantari S. (2012). Insecticied effect of silver and zinc nanoparticles against *Aphis nerii* Boyer of fonsco-lombe (Hemiptera: Aphididae). Chil. J. Agric. Res..

[56] Singh A.K., Raykar V.S. (2008). Microwave synthesis of silver nanofluids with polyvinylpyrrolidone (PVP) and their transport prop-erties. Coll. Polym. Sci..

[57] Gintenreiter S., Ortel J., Nopp H.J. (1993). Bioaccumulation of cadmium, lead, copper, and zinc in successive developmental stages of *Lymantria dispar* L. (Lymantriidae, Lepid)—A life cycle study. Arch. Environ. Contam. Toxicol..

[58] Kramarz P., Kafel A. (2003). The respiration rate of the beet armyworm pupae (*Spodoptera exigua*) after multigeneration intoxication with cadmium and zinc. Environ. Pollut..

[59] Stone D., Jepson P., Laskowski R. (2002). Trends in detoxification enzymes and heavy metal accumulation in ground beetles (Cole-optera: Carabidae) inhabiting a gradient of pollution. Comp. Biochem. Physiol. Part C Toxicol. Pharmacol..

[60] Ballan-Dufrancais C. (2002). Localization of metals in cells of pterygote insects. Microsc. Res. Tech..

[61] Vivekanandhan P., Karthi S., Shivakumar M.S., Benelli G. (2018). Synergistic effect of entomopathogenic fungus *Fusarium oxysporum* extract in combination with temephos against three major mosquito vectors. Pathog. Glob. Health.

[62] Pigino G., Migliorini M., Paccagnini E., Bernini F., Leonzio C. (2005). Fine structure of the midgut and Malpighian papillae in *Campodea* (*Monocampa*) *quilisi* Silvestri, 1932 (Hexapoda, Diplura) with special reference to the metal composition and physi-ological significance of midgut intracellular electron-densegranules. Tissue Cell.

[63] Thabet A.F., Boraei H.A., Galal O.A., El-Samahy M.F., Mousa K.M., Zhang Y.Z., Tuda M., Helmy E.A., Wen J., Nozaki T. (2021). Silica nanoparticles as pesticide against insects of different feeding types and their non-target attraction of predators. Sci. Rep..

[64] Vivekanandhan P., Swathy K., Alford L., Pittarate S., Subala S.P.R.R., Mekchay S., Elangovan D., Krutmuang P. (2022). Toxicity of *Metarhizium flavoviride* conidia virulence against *Spodoptera litura* (Lepidoptera: Noctuidae) and its impact on physiological and biochemical activities. Sci. Rep..

[65] Anreddy R.N.R. (2018). Copper oxide nanoparticles induces oxidative stress and liver toxicity in rats following oral exposure. Toxicol. Rep..

[66] Naz S., Gul A., Zia M. (2020). Toxicity of copper oxide nanoparticles: A review study. IET Nanobiotechnol..

[67] Assadian E., Zarei M.H., Gilani A.G., Farshin M., Degampanah H., Pourahmad J. (2018). Toxicity of copper oxide (CuO) nanoparticles on human blood lymphocytes. Biol. Trace Elem. Res..

[68] Karlsson H.L., Cronholm P., Gustafsson J., Moller L. (2008). Copper oxide nanoparticles are highly toxic: A comparison between metal oxide nanoparticles and carbon nanotubes. Chem. Res. Toxicol..

